# Post-surgery anxiety and depression in prostate cancer patients: prevalence, longitudinal progression, and their correlations with survival profiles during a 3-year follow-up

**DOI:** 10.1007/s11845-020-02417-x

**Published:** 2021-01-07

**Authors:** Su Hu, Li Li, Xiaoling Wu, Zhengqing Liu, Adan Fu

**Affiliations:** Department of Intensive Care Unit, The Central Hospital of Wuhan, Tongji Medical College, Huazhong University of Science and Technology, 26 Shengli Street, Wuhan, 430014 China

**Keywords:** Anxiety, Depression, Disease-free survival, Overall survival, Prostate cancer

## Abstract

**Background:**

Anxiety and depression are more frequent in cancer patients than general population and may be correlated with cancer prognosis; however, their value in prostate cancer patients is largely unknown. We aimed to evaluate prevalence of anxiety and depression in prostate cancer survivors post the surgeries, and their correlations with patients’ disease-free survival (DFS) and overall survival (OS).

**Methods:**

A hundred and ninety-four patients with prostate cancer who underwent radical prostatectomy were enrolled. After discharged from hospital, patients were assessed for post-surgery anxiety and depression every 3 months using Zung Self-rating Anxiety/Depression Scale (SAS/SDS) for a total of 36 months. In addition, disease conditions, DFS, and OS were also documented.

**Results:**

SAS score (*P* < 0.001), anxiety rate (*P* = 0.004), SDS score (*P* < 0.001), and depression rate (*P* < 0.001) gradually elevated from baseline to month 36 in prostate cancer patients. Anxiety at baseline (*P* = 0.009) and anxiety at 3 years (*P* = 0.017) were correlated with worse DFS, and anxiety at baseline (*P* = 0.009) was also correlated with shorter OS in prostate cancer patients. Furthermore, depression at baseline (*P* = 0.005) and depression at 2 years (*P* = 0.008) were associated with unfavorable DFS, and depression at baseline (*P* = 0.001), 1 year (*P* = 0.025), and 2 years (*P* = 0.008) were associated with worse OS in prostate cancer patients. Moreover, multivariate Cox’s proportional hazards regression analysis elucidated that depression at baseline (*P* = 0.027) was an independent predictive factor for shorter DFS in prostate cancer patients.

**Conclusion:**

Anxiety and depression both gradually deteriorate, and they correlate with unfavorable survival profile in prostate cancer patients after radical prostatectomy.

## Introduction

Anxiety and depression have long been the most common mental disorders that impact on approximately 6–7% of the worldwide population, and they often coexist in the same individual [1, 2]. These mental disorders not only sabotage psychiatric health but also result in physical dysfunction, such as increasing cardiovascular disease risk and cause fatigue in affected individuals [3, 4]. In the past decades, anxiety and depression are more and more noted in cancer patients as they tend to have obviously increased prevalence in cancer patients than in the general populations [5, 6]. The mechanism underlying the occurrence of anxiety and depression in cancer patients is complex. Nowadays, several possible mechanisms including the change of biopsychosocial status, some anti-tumor drugs, and neuron affected tumors have been implied [7–9]. However, although anxiety and depression present with a relatively high prevalence in cancer patients, very limited patients can obtain proper intervention, what’s worse is that these two mental disorders are increasingly reported to be correlated with worse prognosis [10, 11].

Among males, prostate cancer is currently the most frequent cancer in over a half of countries worldwide [12]. Prostate cancer treatments are categorized by the disease condition; for localized patients, the first line methods are prostatectomy and radio-therapy, and for metastatic patients, hormone therapy and chemotherapy are the most frequently applied methods in practice [13, 14]. Most of the patients with prostate cancer present with a favorable survival profile; therefore, the number of prostate cancer survivors after the surgeries is very considerable; although survived from the disease, patients have to confront various issues that often impairs their health status and quality of life, such as the fear of relapse and decrease of physical function [15, 16]. Recently, there have been reports of the development of anxiety and depression in prostate cancer survivors, which is not surprising in view of the incidence of these two mental disorders in other cancers [17], while the impact of anxiety and/or depression on prostate cancer patients’ clinical outcome remains ambiguous, and corresponding studies are very few.

Therefore, we investigated the prevalence of anxiety and depression in prostate cancer survivors post the surgeries, and their correlations with patients’ disease-free survival (DFS) as well as overall survival (OS) in this current study.

## Methods

### Patients

From January 2015 to December 2016, 194 patients with prostate cancer who underwent radical prostatectomy in our hospital were consecutively enrolled in this study. The eligible criteria for enrollment were as follows: (1) confirmed diagnosis of primary prostate cancer based on histopathological verification of adenocarcinoma in prostate biopsy cores; (2) age ≥ 18 years; (3) underwent radical prostatectomy; (4) able to understand the study consents and complete the anxiety and depression assessments; (5) willing to comply with follow-up schedule. Patients with following conditions were not included in the study: (1) known other serious mental disorders (excepting anxiety and depression); (2) severe cognitive impairment; (3) history of severe neurological diseases or neurodegenerative disease; (4) complicated with other cancers. All patients signed the informed consents before enrollment. The Institutional Review Board of our hospital approved the current study before initiation.

### Baseline data collection and assessment

Patients’ clinical characteristics, including demographics (age, gender, education duration, marry status, employment status before surgery, and smoke status), comorbidities (hypertension, hyperlipidemia, diabetes, and chronic kidney disease (CKD)), and disease features (prostate-specific antigen (PSA) level, Gleason score, pathological T stage, pathological N stage, surgical margin status), were documented in case report forms on the day of hospital discharge that was defined as baseline (M0). Meanwhile, assessment of anxiety and depression using Zung Self-rating Anxiety/Depression Scale (SAS/SDS) was performed at that time. The assessment was performed by filling the questionnaire by the patients themselves guided by the doctors or nurses, who were blinded to the clinical features of each patient. In detail, before assessment, investigator provided instructions about how to fill in the scales, then patients were required to complete the SAS and SDS by themselves.

### Follow-up and assessment

After discharged from hospital, patients were instructed to return to hospital for assessment of anxiety and depression every 3 months (at month 3 (M3), M6, M9, M12, M15, M18, M21, M24, M27, M30, M33, and M36). The trimonthly assessment was continuously performed for a total of 36 months, and the SAS and SDS scores were calculated at each time point. For patients who lost follow-up, the last assessed SAS and SDS scores were used as subsequent missing scores. Besides, in each clinic visit, disease conditions and survival status of patients were documented for evaluation of disease-free survival (DFS) and overall survival (OS).

### Anxiety and depression definitions by SAS and SDS

There were 20 items in SAS and SDS. Each item was scored as 1–4 points individually, resulting in a 20–80 raw score. The standard score was calculated by multiplying raw scores by 1.25; as a result, the final standard score was ranging from 25~100 points. The anxiety was defined as SAS score ≥ 50; similarly, the depression was defined as SDS score ≥ 50 [18, 19].

### Statistical analysis

Data were presented as mean with standard deviation (M ± SD), median with interquartile range (IQR), or number with percentage (no. (%)). The data distribution was assessed by the Kolmogorov-Smirnov test. Variation tendency of SAS and SDS score was determined by analysis of variance (ANOVA) followed by the Dunnett test for repeated measurements. Variation tendency of anxiety and depression rate was determined by chi-square test for trend. DFS was calculated from the date of surgery to the date of disease relapse, or patients’ death; OS was calculated from the date of surgery to the date of patients’ death. Both DFS and OS were displayed by Kaplan-Meier curves, and the comparison of DFS and OS between different subjects was determined by Log-rank test. All potential factors related to DFS or OS were included in conditional multivariate Cox’s proportional hazards regression with forward stepwise method to analyze the independent factors. Statistical significance was set at *P* value < 0.05. SPSS 22.0 statistical software (IBM, Chicago, IL, USA) and GraphPad Prism 7.02 (GraphPad Software Inc., San Diego, California, USA) were applied for statistical data processing and graphs plotting.

## Results

### Characteristics of prostate cancer patients

Among 194 prostate cancer patients in this study, the mean age was 62.5 ± 9.0 years (Table 1). The median of education duration was 9.0 (6.0–12.0) years, then the numbers of patients with a marry status of married and single/divorced/widowed were 65 (33.5%) and 129 (66.5%), respectively. In addition, there were 128 (66.0%) employed patients and 66 (34.0%) unemployed patients before surgery. The number of smokers was 84 (43.3%). In terms of disease features, the numbers of patients with PSA level ≤ 10 ng/ml, 10–20 ng/ml, and ≥ 10–20 ng/ml were 44 (22.7%), 107 (55.2%), and 43 (22.2%), respectively. There were 44 (22.7%) patients who had Gleason score ≤ 6, 107 (55.2%) patients who had Gleason score of 7, and 43 (22.2%) patients who had Gleason score ≥ 8. And the numbers of patients who had a negative surgical margin and patients who had a positive surgical margin were 160 (82.5%) and 234 (17.5%), respectively. The other detailed information regarding comorbidities and disease features could be seen in Table 1.

**Table 1:** Clinical characteristics of patients

Items	Prostate cancer patients (*N* = 194)
Demographics	
Age (years), M ± D	62.5 ± 9.0
Education duration (years), median (IQR)	9.0 (6.0–12.0)
Marry status, no. (%)	
Married	129 (66.5)
Single/divorced/widowed	65 (33.5)
Employment status before surgery, no. (%)	
Employed	66 (34.0)
Unemployed	128 (66.0)
Smoker, no. (%)	84 (43.3)
Comorbidities	
Hypertension, no. (%)	83 (42.8)
Hyperlipidemia, no. (%)	45 (23.2)
Diabetes, no. (%)	35 (18.0)
CKD, no. (%)	32 (16.5)
Disease features	
PSA level, no. (%)	
≤ 10 ng/ml	46 (23.7)
10–20 ng/ml	100 (51.5)
≥ 10–20 ng/ml	48 (24.8)
Gleason score, no. (%)	
≤ 6	44 (22.7)
7	107 (55.2)
≥ 8	43 (22.1)
Pathological T stage, no. (%)	
pT2	112 (57.7)
pT3	76 (39.2)
pT4	6 (3.1)
Pathological N stage, no. (%)	
pN0	140 (72.2)
pN1	54 (27.8)
Surgical margin status, no. (%)	
Negative	160 (82.5)
Positive	34 (17.5)

*M ± SD*, mean ± standard deviation; *IQR*, interquartile range; *CKD*, chronic kidney disease; *PSA*, prostate-specific antigen

### Anxiety and depression in prostate cancer patients

**Fig. 1:**
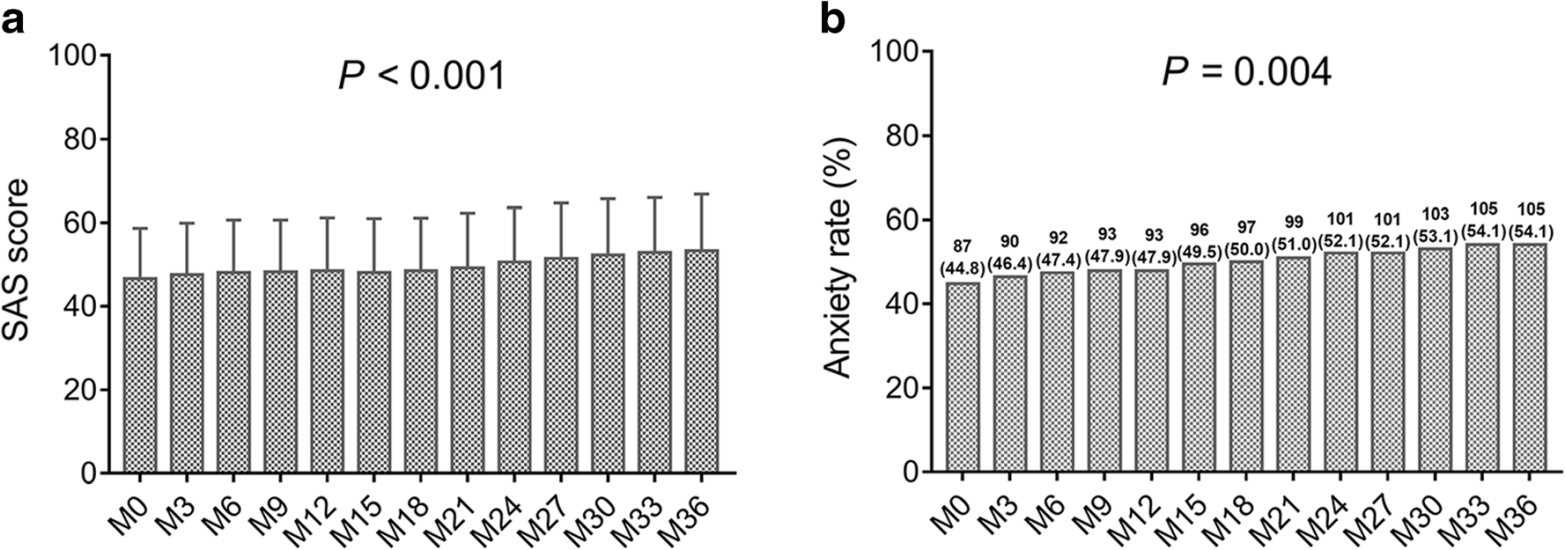
SAS score and anxiety rate at each follow-up. The SAS score at each follow-up (**a**), and anxiety rate at each follow-up (**b**) in prostate cancer patients after surgery. SAS, Zung Self-rating Anxiety scale

**Fig. 2:**
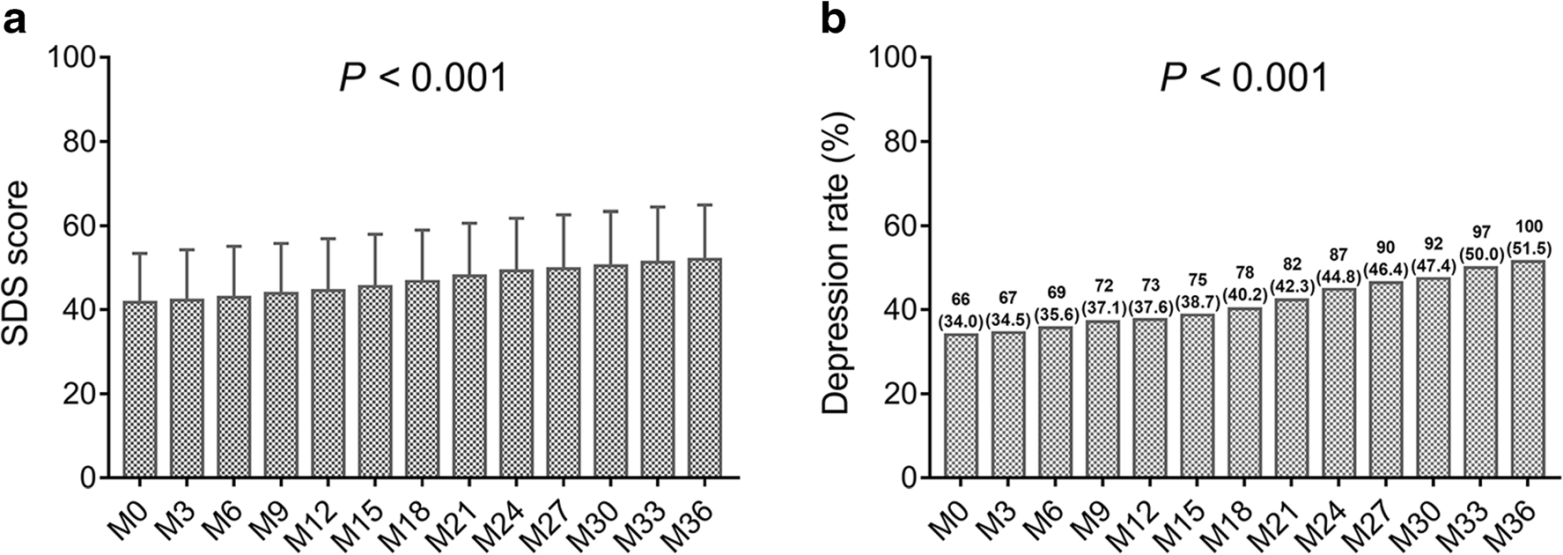
SDS score and depression rate at each follow-up. The SDS score at each follow-up (**a**), and depression rate at each follow-up (**b**) in prostate cancer patients after surgery. SDS, Zung Self-rating Depression scale

At M0, the anxiety rate and depression rate in prostate cancer patients after radical prostatectomy were 44.8% and 34.0%, respectively. From M0 to M36, in terms of anxiety, the SAS score (*P* < 0.001) (Fig. 1a) and anxiety rate (*P* = 0.004) (Fig. 1b) were gradually increased in prostate cancer patients. In regard to depression, the SDS score (*P* < 0.001) (Fig. 2a) and depression rate (*P* < 0.001) (Fig. 2b) were also constantly elevated, and the increase of depression rate numerically exceeded that of anxiety rate. These data indicated that anxiety and depression both became increasingly worse in prostate cancer survivors after radical prostatectomy.

### Difference in survival between patients with anxiety and patients without anxiety

**Fig. 3:**
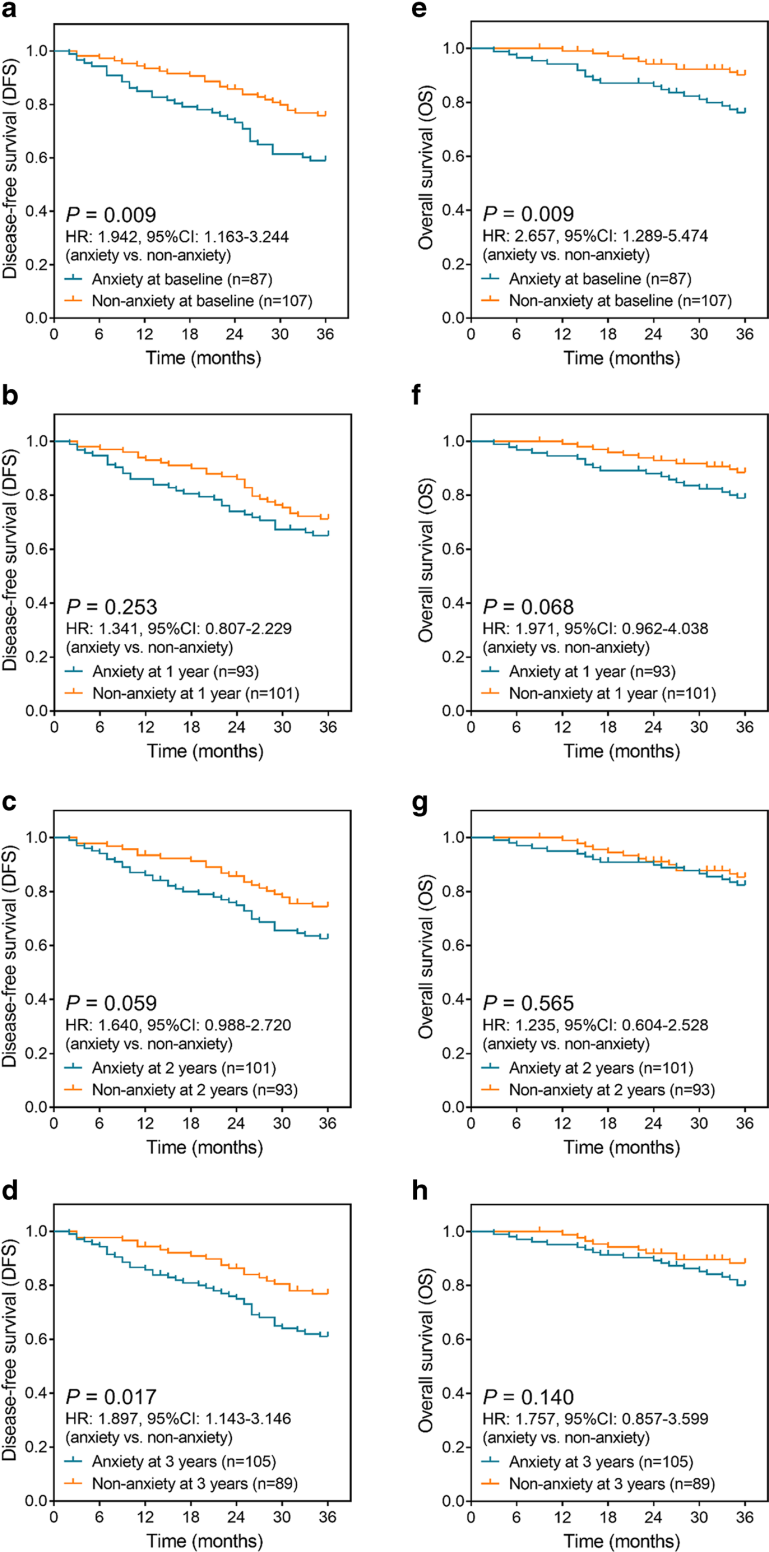
Anxiety correlated with worse DFS and OS. Difference of DFS in prostate cancer patients with anxiety and patients without anxiety at baseline (**a**), 1 year (**b**), 2 years (**c**), and 3 years (**d**); difference of OS in prostate cancer patients with anxiety and without anxiety at baseline (**e**), 1 year (**f**), 2 years (**g**), and 3 years (**h**). DFS, disease-free survival; OS, overall survival; HR, hazard ratio; 95% CI, 95% confidence interval

Anxiety at baseline (*P* = 0.009) (Fig. 3a) and anxiety at 3 years (*P* = 0.017) (Fig. 3d) were correlated with worse DFS in prostate cancer survivors post the surgeries, while anxiety at 1 year (*P* = 0.253) (Fig. 3b) or anxiety at 2 years (*P* = 0.059) (Fig. 3c) was not correlated with DFS. As for OS, anxiety at baseline (*P* = 0.009) (Fig. 3e) associated with shorter OS in prostate cancer survivors post the surgeries, but anxiety at 1 year (*P* = 0.068) (Fig. 3f), anxiety at 2 years (*P* = 0.565) (Fig. 3g), or anxiety at 3 years (*P* = 0.140) (Fig. 3h) was not associated with OS. These results suggested that the survival profile was worse in prostate cancer patients with anxiety.

### Difference in survivals between patients with depression and patients without depression

**Fig. 4:**
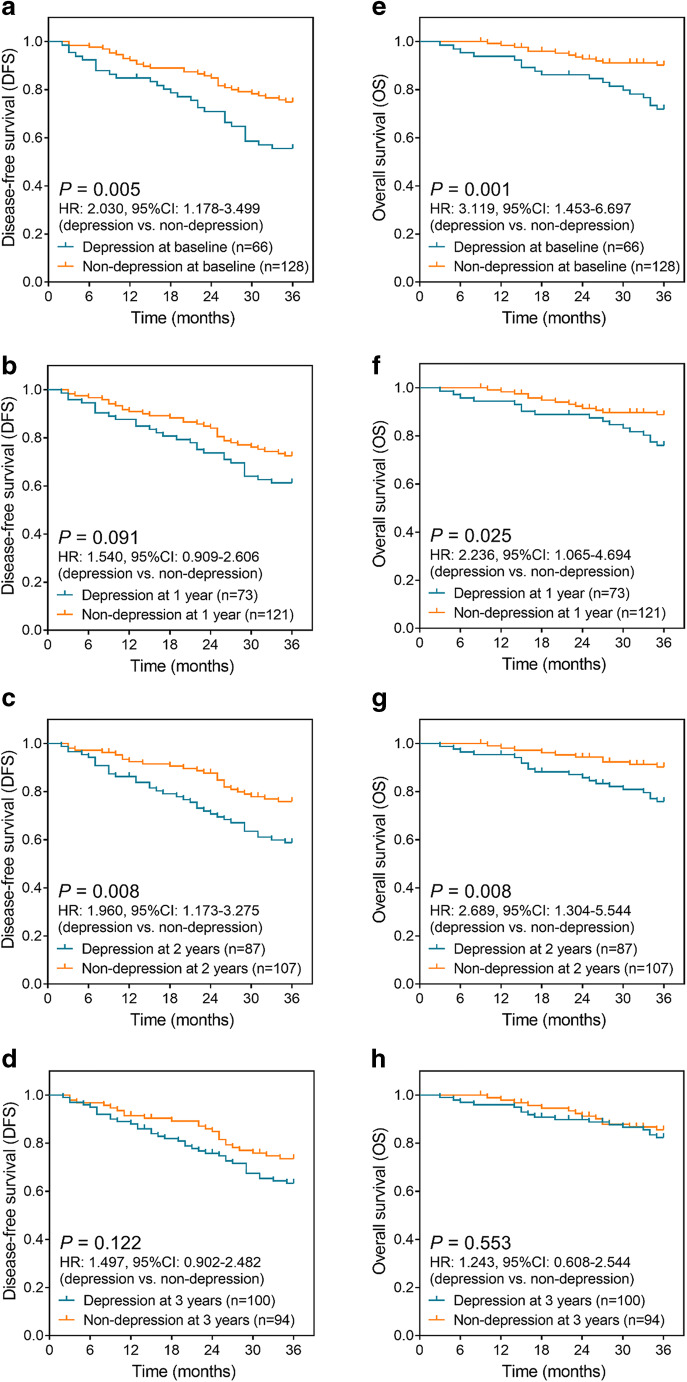
Depression correlated with worse DFS and OS. Difference of DFS between prostate cancer patients with depression and patients without depression at baseline (**a**), 1 year (**b**), 2 years (**c**), and 3 years (**d**); difference of OS between prostate cancer patients with depression and patients without depression at baseline (**e**), 1 year (**f**), 2 years (**g**), and 3 years (**h**). DFS, disease-free survival; OS, overall survival; HR, hazard ratio; 95% CI, 95% confidence interval

Depression at baseline (*P* = 0.005) (Fig. 4a) and depression at 2 years (*P* = 0.008) (Fig. 4c) were correlated with unfavorable DFS in prostate cancer survivors post the surgeries, while depression at 1 year (*P* = 0.091) (Fig. 4b) or depression at 3 years (*P* = 0.122) (Fig. 4d) was not associated with DFS. Moreover, depression at baseline (*P* = 0.001) (Fig. 4e), depression at 1 year (*P* = 0.025) (Fig. 4f), and depression at 2 years (*P* = 0.008) (Fig. 4g) were associated with wore OS, but depression at 3 years (*P* = 0.553) (Fig. 4h) was not correlated with OS. These implied that the survival profile was unfavorable in prostate cancer patients with depression.

### Analyses of independent factors related to survival

Furthermore, multivariate Cox’s proportional hazards regression analysis disclosed that depression at baseline (*P* = 0.027) (HR = 1.796) was a factor that could independently predict shorter DFS in prostate cancer patients after radical prostatectomy, besides, the other independent predictive factors for worse DFS were higher Gleason score (*P* = 0.013) (HR = 1.638) and positive surgical margin (*P* = 0.040) (HR = 1.828) (Table 2). In addition, regarding OS, higher Gleason score (*P* = 0.014) (HR = 2.007), higher pathological T stage (*P* = 0.001) (HR = 2.630), and positive surgical margin (*P* = 0.003) (HR = 3.134) were independent predictive factors for worse OS (Table 3). These findings suggested that depression at baseline was independently correlated with an elevated risk of relapse in prostate cancer patients.

**Table 2:** Multivariate Cox’s proportional hazards regression analysis of factors related to DFS

Factors	Multivariate Cox’s proportional hazards regression analysis					
	B	SE	Wald	*P* value	HR	95% CI (lower-upper)
Depression at baseline	0.586	0.265	4.890	0.027	1.796	1.069–3.018
Higher Gleason score	0.493	0.198	6.181	0.013	1.638	1.110–2.417
Positive surgical margin	0.603	0.294	4.221	0.040	1.828	1.028–3.251

*DFS*, disease-free survival; *SE*, standard error; *HR*, hazards ratio; *CI*, confidence interval

**Table 3:** Multivariate Cox’s proportional hazards regression analysis of factors related to OS

Factors	Multivariate Cox’s proportional hazards regression analysis					
	B	SE	Wald	*P* value	HR	95% CI (lower-upper)
Higher Gleason score	0.696	0.284	6.019	0.014	2.007	1.150–3.500
Higher pathological T stage	0.967	0.303	10.207	0.001	2.630	1.453–4.760
Positive surgical margin	1.142	0.379	9.063	0.003	3.134	1.490–6.592

*OS*, overall survival; *SE*, standard error; *HR*, hazards ratio; *CI*, confidence interval

## Discussion

It is relatively established that anxiety and depression are more likely to occur in cancer patients than in healthy population, with reported prevalence of clinically documented anxiety and depression at approximately 20% and 10% according to a previous meta-analysis [5]. While for prostate cancer, neither the epidemiology of anxiety and depression nor the correlation of anxiety and depression with patients’ outcome is well investigated. Therefore, we conducted this study, and found in prostate cancer survivors post the surgeries the following: (1) prevalence of anxiety and depression post the surgeries was 44.8% and 34.0%, respectively; then anxiety and depression both gradually worsened throughout the 3-year follow-up duration. (2) Anxiety and depression both correlated with worse DFS and OS, and baseline depression was an independent predictive factor for unfavorable DFS.

Throughout the long follow-up time of 3 years in the prostate cancer survivors post the surgeries, we found that the prevalence of anxiety and depression was 44.8% and 34.0%, respectively, and the score and prevalence of anxiety and depression constantly increased from M0 to M36, which obviously caught our attention of the need in psychiatric management. In the previous studies of other cancers, anxiety and depression are also abundantly reported. Such as, a study reports that the prevalence of depressed symptoms assessed by Patient Health Questionnaire-2 (PHQ-2) scale and anxious symptoms assessed by Generalized Anxiety Disorder-2 (GAD-2) scale in breast cancer patients are 38.2% and 32.2%, respectively [20]. And a register-based study that enrolls 3370 working age cancer survivors (who are diagnosed with cancer 25–55 years before) illuminates that 40% of the participants present with moderate to high anxiety score (assessed by German version of HADS scale) [21]. Additionally, a study with a large sample size of 29,366 women diagnosed with breast or other genital organ cancer demonstrates that there are 7994 patients with confirmed depression or anxiety, the incidence of anxiety or depression in breast cancer patients is 8.8 every 100 person-years, and in patients with other genital organ cancer is 5.9 per 100 person-years [22]. As for the findings in our study, they could be explained by the following aspects. First, stress is the predominant cause of anxiety and depression, and coping with prostate cancer, its clinical symptoms, and treatment certainly result in increased stress in prostate cancer patients, which increases the risk of developing mood disorders including anxiety and depression. Second, studies report that the change of testosterone level and androgen deprivation therapy may also play a role in the development of anxiety and depression in prostate cancer patients [23]. Third, radical prostatectomy normally results in a reduced quality of prostate cancer patients’ domestic life, and may also damage patients’ self-esteem; these all constantly contribute to the developments of anxiety and depression. Additionally, we would like to note that other scales may be needed to assess the anxiety and depression in our patients; however, it was difficult to apply another scale since the data in our study was retrospectively analyzed.

In this study, we also discovered that post-surgery anxiety and depression are associated with worse survival profile, and post-surgery depression could independently predict worse DFS in prostate cancer patients. Previous studies all indicate that anxiety and depression are not uncommon in cancer patients; for this reason, many clinicians are trying to reveal some veiled mechanisms. A study elucidates that depression plays a role in enhancing the progression of hepatocellular carcinoma in mice via increasing programmed death 1 (PD-1) level regulated by glucocorticoids in tumor infiltrating natural killer (NK) cells [24]. Another study reveals that depression correlates with worse clinical outcome in gastric cancer patients, and the in vivo and in vitro experiments disclose that gastric cancer–related depression involves the participation of reactive oxygen species via the ABL1-modulated inflammatory pathway [25]. In addition, a previous study illustrates that anxiety and depression play a role in mediating the correlation of chemotherapy-induced peripheral neuropathy with fatigue in colorectal cancer patients; however, this study also states that this needs more experimental evidence to validate [26]. Furthermore, there are already findings regarding cellular mechanisms of the harmful impact of anxiety and depression on prostate cancer patients. For instance, in mouse model of prostate cancer, depression is illuminated to increase the infiltration of myeloid cells and interleukin-6 (IL-6) expressions via mediating sympathetic neuropeptide Y (NPY) signaling pathway and subsequently enhances the tumor growth [27].

And in the clinical setting, there are also studies about the pejorative role of anxiety and depression in cancer patients’ prognosis. For example, a previous study reveals that in oropharynx cancer patients, the self-report depression independently correlates with unfavorable OS and DFS, which is partially similar to our results [28]. In accordance with this, another study reports that in postoperative non-small cell lung cancer patients, post-surgery anxiety predicts shorter OS, and post-surgery anxiety and depression both predict less prolonged DFS [29]. In terms of our results, we tried to explain them by the following possible reasons. First, prostate cancer patients are more likely to have a low adherence to therapies, and this could contribute to a worse treatment efficacy and thus a worse prognosis [30]. Second, anxiety and depression also induce many physical symptoms, such as fatigue, insomnia, and change of weight as well as appetite; these all contribute to a worse physical function and may interfere with the physical health of prostate cancer patients [31, 32]. Third, anxiety and depression may aggregate the disease in prostate cancer patients via affecting several biological processes or pathways, such as regulating the tumor infiltrating NK cells and reactive oxygen species [24–27]. In the future, the status of anxiety and depression may be useful in optimizing the prognosis prediction in surgical prostate cancer patients; however, this should be validated by more high-quality trials or large-scale cohort studies.

Furthermore, there were a few limitations we liked to discuss. First, we only included surgical prostate cancer patients, which indicated that our findings were not applicable in patients who were not suitable for surgery. Second, we would like to enroll more patients in the future study due to that the sample size in this study was comparatively small, and this may result in an insufficient statistical power.

Altogether, anxiety and depression both gradually deteriorate, and they correlate with unfavorable survival profile in prostate cancer patients after radical prostatectomy.

## References

[1] Kessler RC, Bromet EJ (2013). The epidemiology of depression across cultures. Annu Rev Public Health.

[2] Baxter AJ, Scott KM, Vos T, Whiteford HA (2013). Global prevalence of anxiety disorders: a systematic review and meta-regression. Psychol Med.

[3] Mal K, Awan ID, Ram J, Shaukat F (2019). Depression and anxiety as a risk factor for myocardial infarction. Cureus.

[4] Polikandrioti M, Tzirogiannis K, Zyga S, Koutelekos I, Vasilopoulos G, Theofilou P, Panoutsopoulos G (2018). Effect of anxiety and depression on the fatigue of patients with a permanent pacemaker. Arch Med Sci Atheroscler Dis.

[5] Mitchell AJ, Chan M, Bhatti H, Halton M, Grassi L, Johansen C, Meader N (2011). Prevalence of depression, anxiety, and adjustment disorder in oncological, haematological, and palliative-care settings: a meta-analysis of 94 interview-based studies. Lancet Oncol.

[6] Steel Z, Marnane C, Iranpour C, Chey T, Jackson JW, Patel V, Silove D (2014). The global prevalence of common mental disorders: a systematic review and meta-analysis 1980-2013. Int J Epidemiol.

[7] Breitbart W, Rosenfeld B, Tobias K, Pessin H, Ku GY, Yuan J, Wolchok J (2014). Depression, cytokines, and pancreatic cancer. Psychooncology.

[8] Jick S, Li L, Gastanaga VM, Liede A (2015). Prevalence of hypercalcemia of malignancy among cancer patients in the UK: analysis of the Clinical Practice Research Datalink database. Cancer Epidemiol.

[9] Wick W, Hertenstein A, Platten M (2016). Neurological sequelae of cancer immunotherapies and targeted therapies. Lancet Oncol.

[10] Bortolato B, Hyphantis TN, Valpione S, Perini G, Maes M, Morris G, Kubera M, Köhler CA, Fernandes BS, Stubbs B, Pavlidis N, Carvalho AF (2017). Depression in cancer: the many biobehavioral pathways driving tumor progression. Cancer Treat Rev.

[11] Haskins CB, McDowell BD, Carnahan RM (2019). Impact of preexisting mental illness on breast cancer endocrine therapy adherence. Breast Cancer Res Treat.

[12] Bray F, Ferlay J, Soerjomataram I, Siegel RL, Torre LA, Jemal A (2018). Global cancer statistics 2018: GLOBOCAN estimates of incidence and mortality worldwide for 36 cancers in 185 countries. CA Cancer J Clin.

[13] Nguyen-Nielsen M, Borre M (2016). Diagnostic and therapeutic strategies for prostate Cancer. Semin Nucl Med.

[14] Komura K, Sweeney CJ, Inamoto T, Ibuki N, Azuma H, Kantoff PW (2018). Current treatment strategies for advanced prostate cancer. Int J Urol.

[15] Howlader NNA, Krapcho M, Miller D, Bishop K, Kosary CL, Yu M, Ruhl J, Tatalovich Z, Mariotto A, Lewis DR, Chen HS, Feuer EJ, Cronin KA (2017). SEER cancer statistics review, National Cancer Institute, SEER web site.

[16] Noonan EM, Farrell TW (2016). Primary care of the prostate cancer survivor. Am Fam Physician.

[17] Nead KT, Sinha S, Yang DD (2017). Association of androgen deprivation therapy and depression in the treatment of prostate cancer: a systematic review and meta-analysis. Urol Oncol.

[18] Gainotti G, Cianchetti C, Taramelli M, Tiacci C (1972). The guided self-rating anxiety-depression scale for use in clinical psychopharmacology. Act Nerv Super (Praha).

[19] Zung WW, Gianturco JA (1971). Personality dimension and the Self-Rating Depression Scale. J Clin Psychol.

[20] Tsaras K, Papathanasiou IV, Mitsi D (2018). Assessment of depression and anxiety in breast cancer patients: prevalence and associated factors. Asian Pac J Cancer Prev.

[21] Inhestern L, Beierlein V, Bultmann JC, Möller B, Romer G, Koch U, Bergelt C (2017). Anxiety and depression in working-age cancer survivors: a register-based study. BMC Cancer.

[22] Jacob L, Kalder M, Kostev K (2017). Incidence of depression and anxiety among women newly diagnosed with breast or genital organ cancer in Germany. Psychooncology.

[23] Dinh KT, Reznor G, Muralidhar V, Mahal BA, Nezolosky MD, Choueiri TK, Hoffman KE, Hu JC, Sweeney CJ, Trinh QD, Nguyen PL (2016). Association of androgen deprivation therapy with depression in localized prostate cancer. J Clin Oncol.

[24] Zhao Y, Jia Y, Shi T, Wang W, Shao D, Zheng X, Sun M, He K, Chen L (2019) Depression promotes hepatocellular carcinoma progression through a glucocorticoids mediated up-regulation of PD-1 expression in tumor infiltrating NK cells. Carcinogenesis. 10.1093/carcin/bgz017 10.1093/carcin/bgz017 30715244

[25] Huang T, Zhou F, Yuan X, Yang T, Liang X, Wang Y, Tu H, Chang J, Nan K, Wei Y (2019). Reactive oxygen species are involved in the development of gastric cancer and gastric cancer-related depression through ABL1-mediated inflammation signaling pathway. Oxidative Med Cell Longev.

[26] Bonhof CS, van de Poll-Franse LV, Vissers PAJ (2019). Anxiety and depression mediate the association between chemotherapy-induced peripheral neuropathy and fatigue: results from the population-based PROFILES registry. Psychooncology.

[27] Cheng Y, Tang XY, Li YX, Zhao DD, Cao QH, Wu HX, Yang HB, Hao K, Yang Y (2019). Depression-induced neuropeptide Y secretion promotes prostate cancer growth by recruiting myeloid cells. Clin Cancer Res.

[28] Shinn EH, Valentine A, Jethanandani A, Basen-Engquist K, Fellman B, Urbauer D, Atkinson E, Yusuf SW, Lenihan D, Woods ML, Kies MS, Sood AK, Carmack C, Morrison WH, Gillenwater A, Sturgis EM, Garden AS (2016). Depression and oropharynx cancer outcome. Psychosom Med.

[29] Huang X, Zhang TZ, Li GH, Liu L, Xu GQ (2020). Prevalence and correlation of anxiety and depression on the prognosis of postoperative non-small-cell lung cancer patients in North China. Medicine (Baltimore).

[30] DiMatteo MR, Hanskard-Zolnierek KB (2011) Impact of depression on treatment adherence and survival from cancer. In: Kissane DW, Maj M, Sartorius N (eds) Depression and cancer, 1st edn. Wiley-Blackwell, p 101–24

[31] Malhi GS, Mann JJ (2018). Depression. Lancet.

[32] Craske MG, Stein MB (2016). Anxiety. Lancet.

